# Cooling devices used to avoid warm ischemia time injury during kidney transplantation. Systematic review and meta-analysis

**DOI:** 10.3389/fmedt.2025.1600784

**Published:** 2025-08-13

**Authors:** Marco A. Arizmendi-Villarreal, Alejandro Diaz Gonzalez-Colmenero, Jorge A. Cantú-Hernández, Javier Sanchez-Maldonado, Gerardo E. Muñoz-Maldonado, Edelmiro Perez-Rodriguez, Homero A. Zapata-Chavira, Rene Rodriguez-Gutierrez, Francisco J. Reyna-Sepulveda

**Affiliations:** ^1^ Department of General Surgery, Hospital Universitario “Dr. José Eleuterio González”, Universidad Autónoma de Nuevo León, Monterrey, Mexico; ^2^ Department of Transplantation, Hospital Universitario “Dr. José Eleuterio González”, Universidad Autónoma de Nuevo León, Monterrey, Mexico; ^3^ Department of Endocrinology, Hospital Universitario “Dr. José Eleuterio González”, Universidad Autónoma de Nuevo León, Monterrey, Mexico; ^4^ Plataforma INVEST Medicina UANL – KER Unit Mayo Clinic (KER Unit México), Universidad Autónoma de Nuevo León, Monterrey, Mexico

**Keywords:** ischemia-reperfusion injury, cold ischemia time, warm ischemia time, kidney transplantation, organ preservation

## Abstract

**Introduction:**

Warm ischemia during kidney transplantation contributes to graft dysfunction. External cooling devices have been developed to preserve graft during anastomosis, with promising results in experimental models. A systematic review and meta-analysis were conducted to evaluate the effectiveness of renal cooling devices.

**Methods:**

A comprehensive search of seven databases was performed from inception to January 6, 2023. Eligible studies were randomized, prospective, and included a control group. Four studies met the inclusion criteria. The protocol was registered in PROSPERO (CRD42023409480).

**Results:**

All studies reported significantly lower reperfusion temperatures in kidneys treated with cooling devices compared to controls. Histological graft injury, showed no statistically significant difference (SMD −0.95; 95% CI −10.74 to 8.83). However, post-transplant urinary output was significantly higher in the cooling device groups (SMD 0.49; 95% CI 0.10 to 0.88).

**Discussion:**

The overall risk of bias across included studies was high. Cooling devices effectively lower graft temperature and may improve early functional outcomes. However, evidence of histological benefit remains inconclusive. Further clinical trials are needed to confirm efficacy and standardize device implementation in human transplantation.

**Systematic Review Registration:**

https://www.crd.york.ac.uk/PROSPERO/view/CRD42023409480, PROSPERO CRD42023409480.

## 1 Introduction

Kidney transplantation is the most frequently performed organ transplant, accounting for 102,090 procedures worldwide and representing 65% of all solid organ transplants (1). Maintaining the function of the transplanted kidney is essential in kidney transplantation. The outcome of graft function is affected by the duration of the transplant, and the type of ischemia, especially in marginal organs, which have a higher rate of developing delayed graft function (2–4).

Ischemia in the organ can be classified into cold ischemia time (CIT), which is the time from exsanguination and immersion in cold solutions for preservation until the graft exits hypothermia. Warm ischemia time (WIT) is recognized in two phases: the first occurs during procurement, and the second develops during the vascular anastomosis in the recipient (5, 6).

It is recommended to limit the WIT to 30 min in order to reduce the risk of interstitial fibrosis, tubular atrophy, delayed graft function, graft failure, patient mortality, and long-term survival outcomes (7–10). In open surgical techniques, vascular anastomosis typically takes approximately 45 min (4). With the introduction of minimally invasive techniques in renal transplant (robotic and laparoscopic), the anastomosis time has been further extended compared to the open technique (11).

There are currently hypothermic technologies to maintain and even improve the condition of the organ prior to transplantation (12). Multiple methods have focused on organ preservation during transport, including advancing perfusion machines. However, the strategies to induce CIT involve direct interaction with either the preservation environment (e.g., cold static storage) or the vascular system (e.g., perfusion machines), making them inapplicable during the transplantation itself (13).

Traditionally, the most common strategy is the installation of ice water around the kidney, which is a rudimentary and ineffective (14). This has triggered a window of opportunity for developing techniques and devices to mitigate warm ischemia during anastomosis. In response, a range of novel cooling devices have emerged, from frozen pads to pumped cooling systems have been designed to reduce tissue injury (4, 5). Given the increasing diversity and clinical interest in these technologies, the aim of this systematic review is to evaluate all the cooling devices available to avoid warm ischemia time injury during kidney transplantation.

## 2 Materials and methods

The protocol for this systematic review and meta-analysis was registered within the International Prospective Register of Systematic Reviews (PROSPERO) on April 25th, 2024 (protocol ID: CRD42023409480), and it is based on the Preferred Reporting Items for Systematic Reviews and Meta-Analysis (PRISMA) statement guidelines (15). The Cochrane Handbook for Systematic Reviews of Interventions, version 6.3.2, was thoroughly followed throughout all stages of the study (16).

### 2.1 Literature search

An experienced investigator screened the potential research studies by running a search strategy from inception to January 6, 2023, in seven electronic databases: MEDLINE, Embase, Web of Science, Scopus, Cochrane Central Register of Controlled Trials, EBSCOhost, and Latin American and Caribbean Health Sciences Literature Database (LILACS). The search strategy involved employing both keywords and free-text terms to explore relevant studies about external cooling devices to preserve a kidney graft during a transplant procedure. Both studies that tested a cooling device (CD) *in vivo* animal models or human patients were included. Terms such as “graft, kidney”, “cooling system”, “cooling apparatus” and “cooling device” were used during the search strategy. The results were complemented by screening reference lists, grey literature, and contacting experts in the field. The full search strategy is presented in Supplementary Material.

### 2.2 Eligibility criteria

Studies with a randomized, interventional, and comparative design were included. For studies conducted with animal models, they were considered eligible if: (1) used swine as animal models, since they have numerous anatomical and functional similarities with humans, (2) maintained the animal subjects under standard conditions, with water and food provided *ad libitum*, (3) average weight for their age, and (4) were submitted to the transplant procedure under an appropriate anesthesia protocol. Common inclusion criteria included: (1) the use of an external cooling device during the kidney transplant in at least one of the study groups, and (2) at least one study group underwent the kidney transplant without the external cooling device, independently of the technique used (standard, robot-assisted, or laparoscopic). Studies were excluded if they had a single-arm design, compared two or more external cooling devices without including a control group, were an ex vivo animal model study, or had no outcomes of interest reported by the authors. We avoid including case-control studies, systematic reviews, meta-analyses, case reports, basic science research, conference abstracts, letters to the editor, and literature reviews. Studies were not excluded by terms of subjects' sex, year of publication, language, study setting, or time frame.

Studies without a control group were excluded to ensure the methodological integrity of the review and allow for valid comparisons of outcomes. The absence of a comparator group limits the ability to isolate the effect of the cooling intervention from confounding variables or natural variability in perioperative care. Including only comparative studies enhances internal validity and facilitates a more accurate assessment of the effectiveness and safety of external cooling devices during kidney transplantation.

### 2.3 Selection process

**Figure 1:**
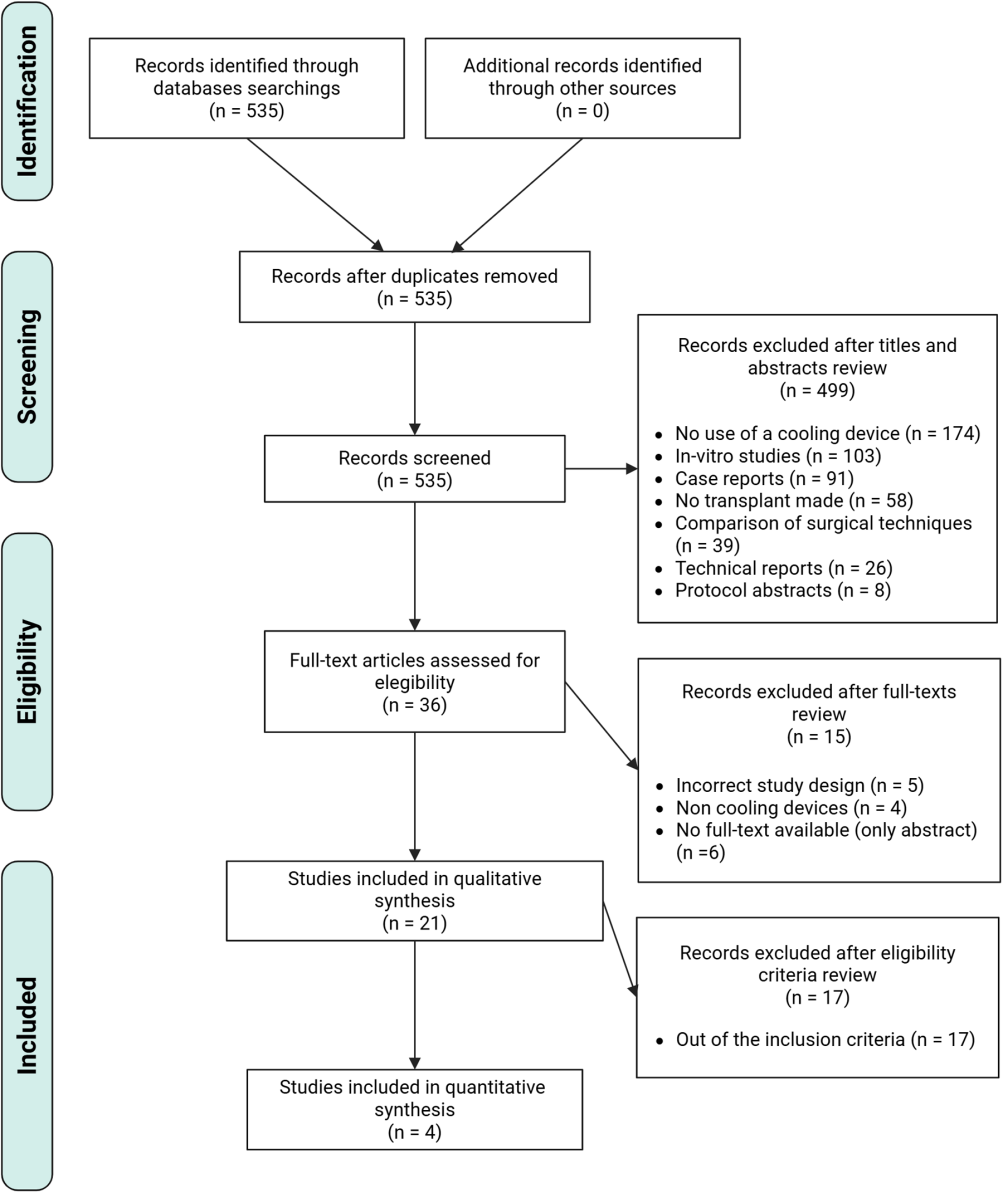
PRISMA flow chart. Overview of all the studies identified, screened and included in the analysis.

Before every screening phase was conducted, a pre-screening pilot phase was carried out among four independent reviewers to adjust their attachment to the selection criteria. Between reviewers' agreement was estimated using the Fleiss' Kappa index, considering a value ≥0.70 as an acceptable cut-off value. The reviewers systematically screened all the studies, independently and blinded, by their abstracts and full text. Any disagreement was solved by consulting an additional reviewer who is an expert in the field (FRS). A detailed graphical representation of the global screening process is shown in (Figure 1).

### 2.4 Data extraction, synthesis, and analysis

A pre-designed extensive database was used for data extraction, based on the specifications described in the Cochrane Handbook for Systematic Reviews (16). For each reference included, descriptive and outcome-related data were extracted. In cases where data was only available in graphic resources (e.g., plots), a web-based digital tool (“WebPlotDigitizer”, https://apps.automeris.io/wpd/) was used to extract quantitative data.

Outcome-related data from primary and secondary outcomes were extracted as Mean ± Standard Deviation (SD) of the scores from the clinical tools used for their assessment. When data was reported as Mean ± Standard Estimate Mean (SEM), SD was obtained by multiplying the SEM by the square root of the sample size n of the group (17):

$$SD=\left(SEM\right)\left(\sqrt{n}\right)$$

Incase a study omitted or unclearly reported an outcome, a letter was sent via email to the corresponding author to make contact and retrieve missing data.

### 2.5 Outcomes of interest

Primary outcomes assessment included objective indicators of the kidney graft's functionality and integrity, such as histological preservation of the nephron, and urinary output after the transplant procedure.

These outcomes were evaluated through:

1. *Goujon's score*: originally evaluated eight histopathological parameters (apical cytoplasm vacuolization, tubular necrosis, tubular dilatation, cell detachment, brush border integrity, intracellular edema, denuded basement membrane, mitochondria integrity) using an ordinal scale from 1 to 5, and consist in the following criteria: 1, no abnormality; 2, mild lesions affecting 10% or less of kidney samples; 3, lesions affecting 25% of kidney samples; 4, lesions affecting 50% of kidney samples; and 5, lesions affecting 75% or more of kidney samples (18). This score, and its modified version, which uses the same scale, but only assesses four histopathological parameters (percentage of glomerular flocculus retraction in Bowman's space, brush border loss, lumina of tubules with cellular debris, and tubular dilatation) are widely used for the histological integrity assessment of renal tissue, specifically after and intervention for its preservation is made.
2. *Urinary output*: Measured as the milliliters per hour produced by the transplanted kidney during the postoperative period of the study subjects.

Secondary outcomes include graft management and indirect preservation indicators, such as surgical time, warm and cold ischemia time, graft temperature during and after the transplant procedure, and time of anastomosis. These were measured in controlled conditions, in a standardized fashion described by each author. All outcomes were extracted as means and standard deviations.

### 2.6 Assessing the risk of bias

Two reviewers independently assessed the risk of bias from the selected studies through one of two possible tools. For studies conducted in animal models, the Systematic Review Centre for Laboratory Animal Experimentation (SYRCLE tool) was used (19, 20). This tool is composed of ten signaling questions, which assess methodological bias in six domains (selection, performance, detection, attrition, reporting, and other type of bias).

### 2.7 Published specifications of experimental cooling devices

We evaluated the studies reporting the use of renal cooling devices, regardless of whether a control group was included, surgical approach, experimental model, and outcome. For each study, we identified and compiled the technical specifications and reported properties of the cooling devices, as described by the inventors or authors. This approach allowed us to compare the various techniques implemented to protect the kidney from warm ischemia. The following characteristics were assessed: device material, manufacturing process, tubing configuration, sterilization methods, type of flow control system, coolant type, temperature monitoring mechanisms, presence of a hilum access window, compatibility with different surgical approaches, and the experimental model in which the device was tested.

### 2.8 Statistical analysis

Analysis of the overall histological preservation was made by pooling the weighted mean treatment effect, based on the standardized mean difference (between-groups Hedge's g) between the Goujon scores (simple or modified) obtained by both intervention and control groups at follow-up. The mean difference was standardized using the pooled SDΔ and corrected for bias due to small sample size. For the interpretation of the effect's magnitude, the following cut-off values were considered (Cohen, 1988): ≥0.8 = large; 0.2–0.5 = medium; and ≤0.2 = small. Since significant heterogeneity between studies was expected due to numerous methodological aspects, a Random-Effects Model was used to pool all overall outcomes.

For the secondary outcomes, the analysis of the weighted mean treatment differences in the evaluation of other functional indicators of the graft (urinary output) and surgical management (surgical time, time of anastomosis, length of cold and warm ischemia periods, and graft temperature at reperfusion) was made through the pooling of the standardized mean difference between measures obtained by both intervention and control groups.

To appraise heterogeneity, Cochran's Q-statistic was employed, adopting a significance threshold of *p* < 0.10. Additionally, the *I*^2^-statistic quantified the proportion of variability attributed to heterogeneity, with values surpassing 50% denoting substantial heterogeneity. Forest plots were used to represent the calculated mean differences graphically. No sensitivity or subgroup analyses were made due to the restricted number of articles used for quantitative analysis. Data analysis was made using meta and dmetar packages in the R statistical version R.4.2.3 software (Posit PBC, Boston, United States).

## 3 Results

We identified 535 studies after the electronic search. After the abstract screening phase, we included 36 studies to evaluate the full manuscript. We excluded 15 studies and finally included 21 studies (3, 4, 11, 21–38) for the qualitative synthesis, and 4 studies (4, 11, 21, 22) included in quantitative synthesis. Further details are shown in (Figure 1) using the PRISMA flow chart. Four studies included animal model. In total, 70 animals were in the quantitative studies.

### 3.1 Study characteristics

A general description of the included studies is presented in (Table 1). All animal models used a porcine model. Three studies included a cooling jacket device with a cold between 0 and 4°C liquid infusion circuit to preserve temperature. One study used an isolation bag preservation (organ pocket), the solutions varied between studies. From the animal studies, 2 used an open technique and 2 used minimal-invasive techniques, including robotic and laparoscopic techniques. Only two animal studies used a direct measurement technique to describe the mean renal temperature after reperfusion. The surgical time in the animal models was similar between groups. Warm ischemia time was reported in all animal studies. Histological outcomes were reported in two of the animal studies using the Goujon score.

**Table 1:** Characteristics of animal model articles.

Author	Cooling device (description)	Preservation solution (*temperature*)	Mean weight (kilograms)	Conditions	*n*	Study groups	Approach	Renal temperature during reperfusion (°C)	Total surgical time (minutes)	WIT/CIT (minutes)	Total time of anastomosis (minutes)	Histological damage					Urine output (ml/h)
												Goujon's score	% RT w/brush border loss	% RT w/cellular debris	% Dilated RT	% Retraction of the flocculus	
Meier, et al. (11)	A watertight double sheath in silicone, continuously perfused by a tubing system with ethanol and methylene blue at 4°C. External and internal thicknesses of 5 and 0.8 mm, respectively.	Institut Georges Lopez-1 (4°C)	50.5 ± 5.9	Standard; water and food provided *ad libitum*	11	Cooling device	RAKT	6.5 ± 3.1	288 ± 55	104 ± 120/135 ± 38	70.4 ± 17.7	10.1 ± 1.5	11.1 ± 6.5	41 ± 26.9	34.4 ± 4.1	29.8 ± 8	225.9 ± 201.3
					6	No cooling device	RAKT	28.7 ± 3.3	263 ± 49	110 ± 70/126 ± 37	74 ± 21.5	13.5 ± 2.4	53 ± 20.1	66.7 ± 35	38.3 ± 8.9	32.1 ± 6	125 ± 136.9
					6	Standard technique	OT	22.5 ± 6.5	258 ± 22	120 ± 120/126 ± 45	48.7 ± 11.2	10.3 ± 2	15.2 ± 6.6	40 ± 32.9	34.2 ± 3.8	31.8 ± 6.8	210.5 ± 230.2
Longchamp, et al. (4)	Double silicone sheet, continuously perfused with 4°C ethanol and methylene blue; 5 and 0,8 mm of external and internal thickness, respectively	Institut Georges Lopez-1 (4°C)	44.7 ± 2.3	Standard; water and food provided *ad libitum*	7	Cooling device	OT	4.3 ± 1.1	353	3.6/129	43	14 ± 1.99	1.9 ± 2.2			19.7 ± 5.1	133.51 ± 281.18
					6	Standard technique	OT	-	382	3.5/128	45	15 ± 3.06	4 ± 3.5			25.1 ± 9.8	56.54 ± 207.96
Zhang, et.al. (21)	Airtight plastic bag, with two silicone or latex tubes, and an end depth less than half of the long; confines double-layer plastic bag jacket with a bicirculating system, perfused with 0–4°C saline continuously Thermal insulation bag	-	45–50	Fasted 12 h before surgery	8	Cooling device	LKT		295.35 ± 43.5	4 ± 0.5/225 ± 19	22 ± 15/46 ± 28						1406 ± 110
					4	Standard technique	LKT		343.35 ± 43	4.5 ± 0.8/275 ± 38	27 ± 15/54 ± 18						NS–
					8	Other cooling device	LKT		317.44 ± 40	4.5 ± 0.5/205 ± 30	26 ± 12/58 ± 16						980 ± 215
Ernst, et.al. (22)		Bretschneider's HTK 500 mL (4°C)	37.9 ± 4.5	Group-housed 12 h day-night cycle preoperative; single-housed postoperative. Feeding 2 times a day with standardized pellets; water 5L each morning and evening.	7	Cooling device	OT		184.17 ± 28.3	43.83 ± 8.47/1444.5 ± 17.75	40.5 ± 10.2						NS
					7	Standard technique	OT		167.57 ± 37	29.86 ± 11/1432.57 ± 10	29.86 ± 11						

#### 3.1.1 Histological outcomes

**Figure 2:**
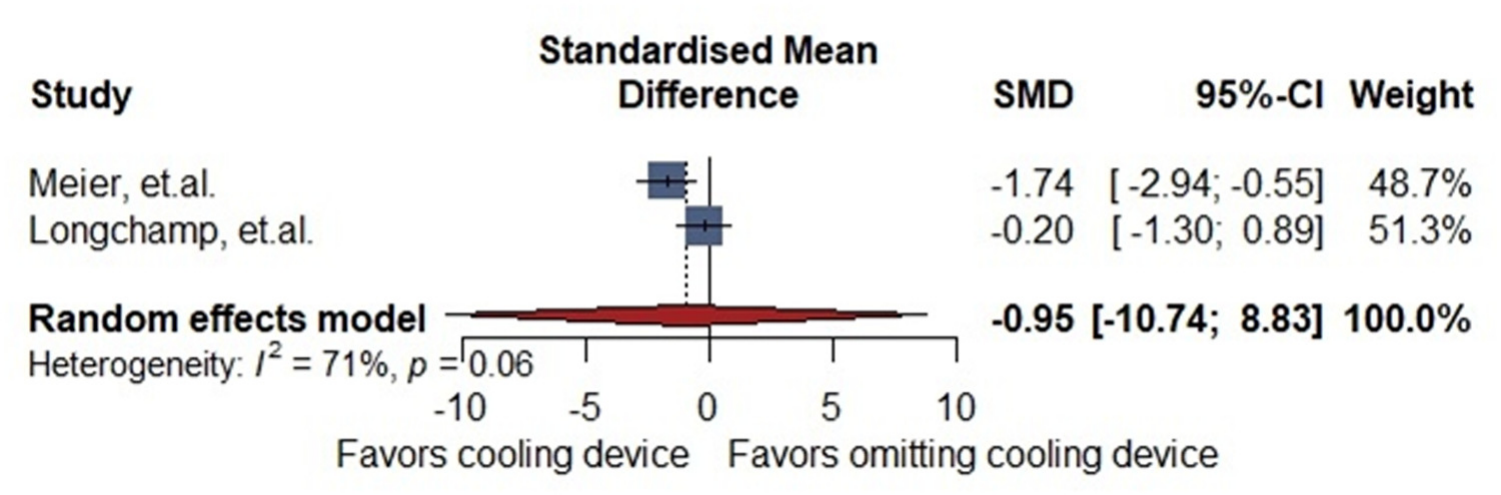
General description of the difference in Goujon score (histopathological damage).

Two animal studies reported the Goujon score (30 porcine models: Cooling device: 18 Non-cooling devices: 12). There was no difference between cooling device and non-cooling device [SMD −0.95 (−10.7–8.8)] with high heterogeneity (I = 71%, *p*. 0.06) (Figure 2).

#### 3.1.2 Urine outcomes

**Figure 3:**
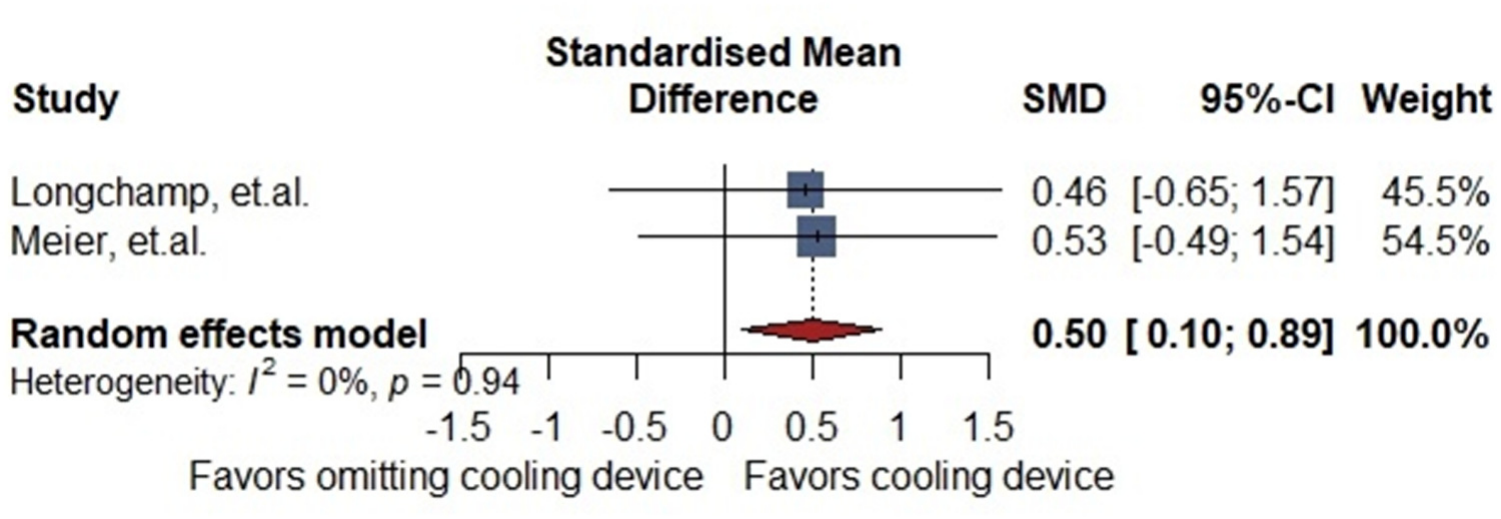
General description of the difference in post-transplant urinary volume.

Two animal studies reported the total urine volume after auto-transplant (30 porcine models: Cooling device: 18 non-cooling devices: 12). The meta-analysis reported a positive difference favoring the cooling device group. [SMD 0.50 (0.1–0.89)] with no heterogeneity (I = 0%, *p* = 0.9) (Figure 3).

### 3.2 Risk of bias

The quality assessment of animal studies is demonstrated (Table 2). Only one of the animal studies generated a random sequence with unknown allocation concealment; moreover, only one study used blinding for the intervention.

**Table 2:** Risk of bias for animal model studies (SYRCLE tool).

Author	Selection bias			Performance bias		Detection bias		Attrition bias	Reporting bias	Other sources of bias
	Sequence generation	Baseline characteristics	Allocation concealment	Random housing	Blinding	Random outcome assessment	Blinding	Incomplete outcome data	Selective outcome reporting	
Meier et al. (11)	Unclear	Yes	No	No	Unclear	No	Yes	Yes	Yes	No
Longchamp et al. (4)		Unclear	No	No		No	Unclear	Yes	Yes	No
	Unclear				Unclear					
Zhang et al. (21)		Yes	No	No		No	Unclear	No	Yes	No
	Unclear				Unclear					
Ernst et al. (22)		Unclear	Unclear		Yes	Yes	Yes	Yes	Unclear.	No
	Yes			Yes						

### 3.3 Published general characteristics of experimental cooling devices

Across the 36 studies reviewed, we identify 21 with a relevant description. A considerable variability in the design and reporting of renal cooling devices. Common materials included plastic polymers (silicone, polyethylene, polylactic acid), with one using aluminum (38). Manufacturing processes were inconsistently detailed; some used 3D printing (31, 35) or homemade sealed bags (21, 31, 34). Silicone or latex tubing was prevalent, but tubing configurations and flow control systems varied, ranging from manual irrigation to peristaltic pumps. Ice-cold saline was the standard coolant. Hilum access windows were frequently included. Devices were tested with open, laparoscopic, or robotic approaches and tested in animal or human models. Renal temperature monitoring during surgery was assessed using different methods across studies (Table 3).

**Table 3:** Published general characteristics of experimental kidney cooling devices.

Author	Device Material	Manufacturing Process	Tubing configuration	Sterilization method	Flow control system	Type of coolant	Temperature monitoring	Hilum access window	Surgical approach compatibility	Experimental model
Forsythe JLR, et al. (23)	Biocompatible plastic, not specified	Two films, with air trapped among the fibers. 8–10 mm in thickness	Not implemented	Not mentioned	Not implemented	Organ pocket with air	Thermocouple	Yes	Ex vivo testing	Swine
Creagh TA, et al. (24)	Polyester laminate (organ pocket)	Polyester laminate which envelope a cross linked polymer distributed in a mineral oil	Not implemented	Not mentioned	Not implemented	Organ pocket with cross linked polymer distributed in a mineral oil	Thermocouple	Yes	Open	Canine
Stephenson RN, et al. (25)	Plastic, not specified	Homemade sealed plastic bag closed with titanium clips 300*280 mm	Not implemented	Not mentioned	Manual irrigation via syringe (120 ml/5 min)	Ice cold saline water (0–1.5°C)	Core and cortical thermometers	Yes	Ex vivo testing	Swine
Herrell SD, et al. (26)	Polymer, not specified	Not mentioned	Cooling tubing, material not specified	Sterilized, method not specified	Tubing pump	Ice cold saline water (3–5°C).	Recorded, method not specified	Yes	Laparoscopic Open	Swine Swine
Colechin ES et al.(27)^a^	Plastic, not specified	Double sheath laparoscopic kidney device	Cooling tubing, material not specified	Not Sterilized	Peristaltic pump (250 ml/min)	Ice cold saline water (0°C).	K-thermocouple	Yes	Laparoscopic	Swine
Planet M, et al. (28)	Rigid biocompatible polyester	Two half-jackets fitted around the graft by means of two magnets.	Not implemented	Not mentioned	Not implemented	Organ pocket with multitherm sponge impregnated with water and a metal mesh	Not recorded	Yes	Open	Swine
Navarro AP, et al. (29)^a^	Plastic, not specified	Double sheath laparoscopic kidney device	Cooling tubing, material not specified	Not Sterilized	Peristaltic pump (250 ml/min)	Ice cold saline water (0°C).	K-thermocouple	Yes	Laparoscopic	Swine
Han X, et al. (30)^e^	Silicon Plastic bag, not specified	Silicon tubes taken from infusers envelop the kidney spirally Common plastic bag 120 * 200 mm	Both devices with silicon tubes	Both sterilized, method not specified	Both with a continue infusion (5–10 ml/min)	Both with ice water (°C not mentioned).	DT-613 Thermal detector	Yes	Laparoscopic	Swine
Kamińska, et al. (3)	Polyethylene bag	The bag consisted of three compartments The middle homing the graft, and two external filled with ice.	Not mentioned	Not mentioned	Not mentioned	Ice cold saline water (°C not mentioned).	Recorded, method not specified	Not mentioned	Open	Human
Meier, et al. (11)^c^	Silicone	Double sheath 0.8 mm inner layer, and 5.0 mm outer layer	Silicone cooling tubing (7 mm OD)	Not mentioned	Tubing pump	Ethanol and methylene blue at 4°C	Thermal probe	Yes	Robotic	Swine
Longchamp, et al. (4)^c^	Silicone	Double sheath 0.8 mm inner layer, and 5.0 mm outer layer	Silicone tubes (7 mm OD)	Not mentioned	Adjustable peristaltic pump	Ethanol and methylene blue at 4°C	Thermal probe	Yes	Open	Swine
Li, et al. (31)^b^	Plastic bag	Homemade sealed plastic bag closed with titanium clip Dimension: 120*200 mm	Silicone or latex tubes	Sterilized, method not specified	Manual irrigation with syringes (250 ml/min)	Ice cold saline water (0–4°C)	JK808 handheld multi-channel temperature measuring	Yes	Minimally Invasive	Human
Territo, et al. (32)	Flexible and elastic polymer	Kidney template from DICOM File Two thin films sealed	Silicone cooling tubing	Not mentioned	Peristaltic pump, activated every 10 min	Ice cold saline water (4°C).	FLIR C2 infrared thermal camera	Yes	Ex vivo testing Open and robotic Open and robotic	Swine Swine Human
Khan T, et al. (33)^d^	Transil® translucent silicone or Polyurethane (organ pocket)	The samples were produced using custom polylactic acid mold tools, fabricated through 3D-Printing.Thickness < 5 mm	Not implemented	Not mentioned	Not implemented	Organ pocket	DS18B20 digital sensor Non-contact thermometer QM7420	Yes	Ex-vivo testing	Swine
Zhang, et al. (21)^b^	Plastic bag	Homemade sealed plastic bag closed with titanium clips or silk 120*200 mm	Silicone or latex tubes	Sterilized, method not specified	Manual irrigation with syringes (200 ml/min)	Ice cold saline water (0–4°C)	DT-613 Thermal detector	Yes	Laparoscopic	Swine
Zhu, et al. (34)^b^	Plastic bag	Homemade sealed plastic bag closed with titanium clips or silk 120*200 mm	Silicone or latex tubes	Sterilized, method not specified	Manual irrigation with syringes (200 ml/min)	Ice cold saline water (0–4°C)	DT-613 Thermal detector	Yes	Laparoscopic	Swine
Cui D, et al. (35)	Polylactic acid	Kidney template from DICOM File for 3D-Printing 1.5 mm inner layer, and 2.0 mm outer layer	IV tubing	70% ethanol for 8 h Plasma hydrogen for 90 min	Continue infusion, not specified	Ice cold saline water (°C not mentioned).	Not recorded	Yes	Laparoscopic	Human
Ernst, et al. (22)	Low-hardness styrenic elastomer gel	Thermal bag-shaped	Not implemented	Not mentioned	Not implemented	Not implemented	FLIR 95 infrared thermal camera	Yes	Ex vivo testing *in vivo*, approach not specified	Swine Swine
Khan T, et al. (36)^d^	Transil® translucent silicone or Polyurethane (organ pocket)	The samples were produced using custom polylactic acid mold tools, fabricated through 3D-Printing.Thickness < 5 mm	Not implemented	Not mentioned	Not implemented	Organ pocket	DS18B20 digital sensor Non-contact thermometer QM7420	Yes	Ex-vivo testing	Swine
Torai, et al. (37)	Silicone or elastomer gel	Bag-shaped organ protector	Not implemented	Not mentioned	Not implemented	Not implemented	K-thermocouple	Yes	Ex-vivo testing	Swine
Dergham, et al. (38)	Food-grade aluminum	Kidney template from DICOM File Food-grade aluminum tubing 3/16 OD, manually positioned in serpentine pattern	Food-grade aluminum	Metal allows sterilization	Manual irrigation (240 ml/min)	Ice cold saline water (4°C)	FLIR TG165 infrared thermal camera	Yes	Ex vivo testing	Swine

^a,b,c,d^ Each number refers to the same experimental prototype evaluated across different studies to validate its safety and effectiveness.

^e^ This study designed and validated two different devices within the same protocol.

## 4 Discussion

Preclinical studies included in this systematic review did not demonstrate significant histological or clinical differences between groups receiving a cooling device and those undergoing standard procedures during kidney transplantation. Nevertheless, in all animal studies utilizing a cooling device, renal surface temperatures during vascular anastomosis were consistently maintained below 20°C, underscoring the device's effectiveness in mitigating warm ischemia (11, 21, 22). To further assess its impact, we conducted two meta-analyses—one evaluating histological injury scores and the other analyzing urine output in swine models. While histological findings did not differ significantly between groups, the use of cooling devices was associated with a notable increase in post-reperfusion urine production, suggesting improved early graft function.

Comparative analyses across surgical approaches—open, laparoscopic, and robotic—revealed no significant differences in temperature, histological, and clinical outcomes demonstrating the usefulness of these devices for minimally invasive transplant techniques. A previous systematic review identified 3 studies using cooling jackets with cold solution flow in robotic and laparoscopic kidney transplants. While these studies reported adequate surface cooling and acceptable anastomotic times, their lack of control groups limits the strength of the evidence and precludes firm conclusions regarding efficacy (14).

Previous experimental data have identified a critical thermal threshold between 15°C and 20°C, beyond which graft function may be compromised. It is well established that for every additional 10 min of exposure to temperatures above this threshold, the risk of delayed graft function (DGF) and acute rejection increases proportionally in human studies (39, 40). Based on the translation potential of this technology, we found only one clinical study that included 46 kidney transplant recipients using a cooling device with an ice-cooled sterile saline solution in an open technique, utilizing a paired-donor allograft model and control group. The cooling device showed a significantly lower rate of detrimental events (delayed graft function and/or acute rejection) as well a higher glomerular filtration rate on day 14 and a greater decrease of MMP9 and LCN2 gene expression, they demonstrated a statistically significant benefit from using a cooling device compared to the control group (3). No statistically significant difference in the main histological features and Remuzzi score with the cooling device and without it (2.4  ±  1.5 vs. 2.6  ±  1.6).

No included study provided a direct comparison between different cooling device designs, and there is currently no evidence supporting the superiority of one configuration over another. The clinical implementation of renal cooling devices faces significant logistical barriers due to the lack of standardization in design, sterilization protocols, and surgical compatibility and minimal time of use per surgery, which complicates routine use. Cost limitations arise from high production expenses, absence of scalable manufacturing, and an unclear cost-benefit ratio given the limited clinical outcome data. From a safety perspective, insufficient reporting on biomaterials, sterilization methods, and adverse events raises concerns about patient risk and regulatory approval. Finally, effectiveness remains uncertain, as no study directly compares devices or provides robust evidence on long-term graft outcomes, hindering the establishment of a superior technique.

To our knowledge, this is the first systematic review focus specifically on purpose-designed cooling devices to prevent warm ischemia in kidney transplants evaluated under standardized experimental conditions with appropriate control arms. In contrast, the review by Andras et al. included studies of a broader array of interventions, including slush ice, cold saline, gauze jackets, and cooling jackets, and most of the studies lacked design specificity or translational potential (14). In contrast, our review exclusively included cooling devices intended for clinical application and supported by preclinical evidence of thermal transfer, histological, and clinical outcomes.

The emergence of robotic and laparoscopic techniques in renal transplantation, while offering numerous surgical advantages, has been associated with prolonged vascular anastomosis times and, consequently, extended warm ischemia periods (41). Cooling devices present a promising and safe intervention to mitigate the adverse impact of this limitation on graft function. Future clinical studies should aim to identify the most effective device configurations and evaluate their application in minimally invasive settings. Moreover, these technologies hold particular promise for high-risk subpopulations predisposed to longer anastomosis times, such as obese recipients or those receiving right-sided donor kidneys (42). To establish the role of cooling devices as a standard of care during vascular anastomosis, robust evidence from randomized controlled trials is essential. One such trial, currently underway, is a single-center study evaluating the Kidney Skin System, registered on ClinicalTrials.gov with a projected two-year duration. The results of this trial may provide critical insight into the clinical utility and standardization of cooling devices in contemporary transplant surgery.

### 4.1 Limitations

This study has several limitations. First, the evidence based on preclinical studies with animal models, which may not be replicated in clinical studies, particularly for the lack of long-term follow-up. While a high number of single-arm studies and case series don't report adverse outcomes using cooling devices, the absence of randomized controlled trials limits the strength of the conclusions that can be drawn. Second, we have excluded a high amount of studies due to the strict criteria of inclusion of studies with the intervention without a control group. This, means that we only obtained a four preclinical studies in animals, limiting the performance of the meta-analysis. Third, the screened records exhibited a high risk of outcome reporting bias, which may impact the reliability of our findings. To mitigate this during the study selection process, we employed a comprehensive and systematic approach based on predefined inclusion criteria. Specifically, we included all studies that reported any clinical or experimental evaluation of cooling devices, irrespective of whether the outcomes were positive, negative, or inconclusive. Additionally, none of the studies have explored the short-term and long-term security profile of the devices including the prolonged anastomosis time, increased risk of wound infection, elevated costs, or other associated complications. Finally, a significant limitation across the included studies is the insufficient reporting of key technical aspects of the cooling devices. Most publications lacked detailed descriptions of the device design, the biomaterials employed, sterilization methods. Also, the high variety of strategies used to monitor thermal transfer at the renal surface. This lack of standardized reporting impairs the ability to critically assess the safety and efficacy of the inventions.

## 5 Conclusion

Our systematic review underscores a critical gap in high-quality, controlled human studies evaluating the efficacy and safety of cooling devices in renal transplantation. Although animal models suggest these devices may enhance early graft function, particularly through increased urine output and protection from warm ischemia, the extrapolation of these findings to clinical practice remains speculative. A major limitation across the literature is the lack of standardized and detailed descriptions regarding device design, functional mechanisms, biomaterials used, and sterilization protocols. This absence of transparency hinders reproducibility, regulatory evaluation, and the development of a consensus process on its implementation. Moreover, the current lack of comparative analyses between device models limits the identification of an optimal design. As minimally invasive techniques continue to extend warm ischemia times, the role of cooling devices may become increasingly critical. The transplantation community must now critically examine if we are ready to embrace these innovations without first demanding standardization, transparency, and evidence of long-term safety.

## Data Availability

The original contributions presented in the study are included in the article/Supplementary Material, further inquiries can be directed to the corresponding author.
