# Supplementary material for: Cooling devices used to avoid warm ischemia time injury during kidney transplantation. Systematic review and meta-analysis
Source: Front Med Technol. 2025 Aug 13;7:1600784. doi: 10.3389/fmedt.2025.1600784 (PMC12380627; doi:10.3389/fmedt.2025.1600784)
Supplement: Supplementary file 1 [file Table1.docx]

Supplementary Material

# Search strategy

“Intraoperative use of external cooling devices in kidney transplantation: protocol for systematic review and meta-analysis of the current literature”

**Keywords implemented:**

("graft, kidney" or "kidney transplant" or "kidney transplant registry" or "renal graft" or "renal transplant" or "transplant, kidney" or "kidney graft") AND ("cooling" or "cooling system" or "cooling apparatus" or "cooling box" or "cooling device" or "cooling filter apparatus")

| Database | Before deduplication | After deduplication |
| --- | --- | --- |
| MEDLINE | 156 | 74 |
| Embase | 135 | 69 |
| Web of Science | 35 | 20 |
| Scopus | 202 | 202 |
| CCRCT | 5 | 5 |
| CDSR | 0 | 0 |
| EBSCOhost | 153 | 142 |
| LILACS | 25 | 22 |
| Total | 535 | |

All searches were run on January 06, 2023.

**EBM Reviews - Cochrane Central Register of Controlled Trials <December 2022>**

**EBM Reviews - Cochrane Database of Systematic Reviews <2005 to January 4, 2023>**

**Embase <1974 to 2023 January 06>**

**Ovid MEDLINE(R) ALL <1946 to January 06, 2023>**

1 exp kidney graft/ 159644

2 ("graft, kidney" or "kidney transplant" or "kidney transplant registry" or "renal graft" or "renal transplant" or "transplant, kidney" or "kidney graft").mp. [mp=ti, ot, ab, fx, sh, hw, kw, tx, ct, tn, dm, mf, dv, kf, dq, bt, nm, ox, px, rx, ui, sy] 162550

3 1 or 2 226975

4 cooling system/ 691

5 ("cooling" or "cooling system" or "cooling apparatus" or "cooling box" or "cooling device" or "cooling filter apparatus").mp. [mp=ti, ot, ab, fx, sh, hw, kw, tx, ct, tn, dm, mf, dv, kf, dq, bt, nm, ox, px, rx, ui, sy] 103493

6 4 or 5 103493

7 3 and 6 281

8 from 7 keep 1-5 5

9 from 7 keep 6-125 120

10 from 7 keep 126-281 156

**SCOPUS 202 document results**

( "graft, kidney"  OR  "kidney transplant"  OR  "kidney transplant registry"  OR  "renal graft"  OR  "renal transplant"  OR  "transplant, kidney"  OR  "kidney graft" )  AND  ( "cooling" or "cooling system"  OR  "cooling apparatus"  OR  "cooling box"  OR  "cooling device"  OR  "cooling filter apparatus" )

**WOS 5 document results**

ALL=(("graft, kidney" or "kidney transplant" or "kidney transplant registry" or "renal graft" or "renal transplant" or "transplant, kidney" or "kidney graft") AND ("cooling" or "cooling system" or "cooling apparatus" or "cooling box" or "cooling device" or "cooling filter apparatus"))

**LILACS 25**

("renal transplant" ) OR ("kidney transplant" ) OR ("kidney transplant registry" ) OR ("renal graft") OR ("renal transplant" ) OR ("transplant, kidney" ) OR ("kidney graft") AND (COOLING)

( renal transplant' or 'kidney transplant OR "kidney transplant registry" OR "renal graft" OR "renal transplant" OR "transplant, kidney" OR "kidney graft" ) AND cooling

**EBSCOhost 233 sin duplicados 153**

| S1 | ("graft, kidney" or "kidney transplant" or "kidney transplant registry" or "renal graft" or "renal transplant" or "transplant, kidney" or "kidney graft") AND ("cooling" or "cooling system" or "cooling apparatus" or "cooling box" or "cooling device" or "cooling filter apparatus") |
| --- | --- |

**Interface**

 - EBSCOhost Research Databases

**Search Screen**

 - Advanced Search

**Database**

 - Academic Search Alumni Edition;Academic Search Ultimate;Alt HealthWatch;America: History and Life with Full Text;Applied Science & Technology Source;Art & Architecture Source;Business Source Complete;Business Source Ultimate;CINAHL Plus with Full Text;Communication Source;Dentistry & Oral Sciences Source;eBook Collection (EBSCOhost);EconLit with Full Text;Education Source;ERIC;Film & Television Literature Index with Full Text;Fuente Académica;Fuente Académica Plus;GreenFILE;Health Business Elite;Historical Abstracts with Full Text;History Reference Center;History Reference eBook Collection;Hospitality & Tourism Complete;Humanities Source;Legal Source;LGBTQ+ Source;Library & Information Science Source;Literary Reference Center;Literary Reference eBook Collection;MAS Reference eBook Collection;MAS Ultra - School Edition;MasterFILE Premier;MasterFILE Reference eBook Collection;MedicLatina;MEDLINE Complete;Newspaper Source Plus;Newswires;OpenDissertations;Political Science Complete;Psychology and Behavioral Sciences Collection;Regional Business News;Religion and Philosophy Collection;Research Starters - Business;Research Starters - Education;Research Starters - Sociology;Science Reference Center;The Serials Directory;Science Reference eBook Collection;SocINDEX with Full Text;SPORTDiscus with Full Text;Textile Technology Complete;Web News;World Politics Review;Points of View Reference Center
